# Association between bacterial finding, antibiotic treatment and clinical course in patients with pharyngotonsillitis: a registry-based study in primary healthcare in Sweden

**DOI:** 10.1186/s12879-021-06511-y

**Published:** 2021-08-09

**Authors:** Jon Pallon, Martin Sundqvist, Mattias Rööst, Katarina Hedin

**Affiliations:** 1 Department of Clinical Sciences in Malmö, Family Medicine, Clinical Research Centre, Lund University, Box 50332, 202 13 Malmö, Sweden; 2 Department of Research and Development, Region Kronoberg, Växjö, Sweden; 3 Department of Laboratory Medicine, Clinical Microbiology, Faculty of Medicine and Health, Örebro University, Örebro, Sweden; 4 Futurum, Region Jönköping County, and Department of Health, Medicine and Caring Sciences, Linköping University, Linköping, Sweden

**Keywords:** Pharyngotonsillitis, *Fusobacterium necrophorum*, Group A streptococci, Aetiology, Primary healthcare, *Streptococcus dysgalactiae* subspecies *equisimilis*

## Abstract

**Background:**

The role of non-group A streptococci and *Fusobacterium* *necrophorum* in pharyngotonsillitis has been disputed and few prospective studies have evaluated the effect of antibiotic treatment. This study uses registry data to investigate the relation between antibiotic prescription for pharyngotonsillitis in primary healthcare and return visits for pharyngotonsillitis, complications, and tonsillectomy.

**Methods:**

Retrospective data were extracted from the regional electronic medical record system in Kronoberg County, Sweden, for all patients diagnosed with pharyngotonsillitis between 2012 and 2016. From these data, two cohorts were formed: one based on rapid antigen detection tests (RADT) for group A streptococci (GAS) and one based on routine throat cultures for β-haemolytic streptococci and *F.* *necrophorum*. The 90 days following the inclusion visit were assessed for new visits for pharyngotonsillitis, complications, and tonsillectomy, and related to bacterial aetiology and antibiotic prescriptions given at inclusion.

**Results:**

In the RADT cohort (n = 13,781), antibiotic prescription for patients with a positive RADT for GAS was associated with fewer return visits for pharyngotonsillitis within 30 days compared with no prescription (8.7% vs. 12%; *p* = 0.02), but not with the complication rate within 30 days (1.5% vs. 1.8%; *p* = 0.7) or with the tonsillectomy rate within 90 days (0.27% vs. 0.26%; *p* = 1). In contrast, antibiotic prescription for patients with a negative RADT was associated with more return visits for pharyngotonsillitis within 30 days (9.7% vs. 7.0%; *p* = 0.01). In the culture cohort (n = 1 370), antibiotic prescription for patients with *Streptococcus dysgalactiae* ssp. *equisimilis* was associated with fewer return visits for pharyngotonsillitis within 30 days compared with no prescription (15% vs. 29%; *p* = 0.03).

**Conclusions:**

Antibiotic prescription was associated with fewer return visits for pharyngotonsillitis in patients with a positive RADT for GAS but with more return visits in patients with a negative RADT for GAS. There were no differences in purulent complications related to antibiotic prescription.

**Supplementary Information:**

The online version contains supplementary material available at 10.1186/s12879-021-06511-y.

## Background

Infectious pharyngotonsillitis can be caused by a wide array of viruses and bacteria, of which *Streptococcus pyogenes* (group A streptococci, GAS) is the most important pathogen and the only one that warrants antibiotic treatment according to most guidelines [1–4]. The indication of antibiotic therapy, however, is confined to reducing symptoms as non-purulent complications of GAS such as rheumatic fever and glomerulonephritis are rare in high-income countries [3] and purulent complications such as peritonsillitis, sinusitis, and media otitis occur in less than 1% of patients [5].

The Sore Throat Guideline Group within the European Society for Clinical Microbiology and Infectious Diseases advocates using the Centor scoring system (one point each for fever, cervical lymphadenitis, tonsillar coating, and absence of cough) [6] to select patients with a higher likelihood of GAS infection (i.e., 3–4 criteria) and considering using a Rapid Antigen Detection Test (RADT) for these patients [3]. Throat cultures are not necessary for routine diagnosis of GAS nor after a negative RADT [3]. Penicillin V, twice or three times daily for 10 days, is the recommended treatment of GAS [3], but should be avoided in patients with Centor score 0–2 as these patients do not seem to benefit from antibiotics [3]. The Swedish Medical Products Agency has adopted this guideline for the most part but stresses that an RADT should only be performed in patients with Centor scores 3–4 as these are the patients who could benefit from antibiotic treatment [1].

In addition to GAS, *Streptococcus dysgalactiae* subspecies *equisimilis* (SDSE), formerly described as large colony group C or G streptococci in the Lancefield classification system [7], has been detected in 9 to 15% of young adults with pharyngotonsillitis [8–10], and the anaerobe *Fusobacterium necrophorum* has been detected in 18–19% of patients with pharyngotonsillitis in primary healthcare (PHC) [11, 12]. Both bacteria, however, are also recovered from healthy controls, and their roles as pathogens in pharyngotonsillitis are still disputed [10–14]. *F.* *necrophorum*, the main pathogen causing the severe but unusual Lemierre’s syndrome [15], has been associated with peritonsillar abscesses [16] and several case reports have described complications following pharyngotonsillitis associated with group C and group G streptococci [3]. Most cases of peritonsillitis, however, are not preceded by a recorded pharyngotonsillitis [17] and few prospective studies have approximated the incidence of complications after an episode of pharyngotonsillitis. Furthermore, no randomised controlled study has shown that antibiotic treatment of pharyngotonsillitis caused by SDSE or *F.* *necrophorum* lowers the complication rate [10, 12].

This study uses registry data to prospectively follow patients with a PHC-recorded pharyngotonsillitis for 90 days and to quantify the incidence of new visits for pharyngotonsillitis, complications, and tonsillectomy in relation to initial aetiology and antibiotic prescription.

## Methods

### Study population and setting

This study was conducted in Kronoberg County in southern Sweden. The Swedish healthcare system is mainly tax-funded and is equally accessible to all inhabitants, with the services decentralised to 21 regional councils. PHC is provided by approximately 1 200 PHC centres (PHCC) dispersed throughout the country. People are encouraged to contact their PHCC before seeking emergency care at hospitals. Therefore, sore throat and other respiratory infections are usually managed by the PHCC.

During the study period (2012–16), the median population in Kronoberg County was 189 292, about 2% of the Swedish population. This population was served by two hospitals and 34 PHCCs, 31 of which participated in the study. The PHCCs were generally open between 08:00 and 17:00, and two out-of-hours centres also served patients between 17:00 and 21:00. In most cases, patients were first assessed over the telephone by a triage nurse, who decided if a physician’s visit was necessary. All visits with a physician required that the physician register a diagnosis code according to the 10^th^ revision of the *International Statistical Classification of Diseases and Related Health Problems* (ICD-10) or its modified Swedish PHC edition (KSH97-P) [18].

This paper is reported following the STROBE statement [19] and the RECORD statement [20].

### Data extraction

Retrospective data for the years 2012–16 were extracted from the regional electronic medical record (EMR) system (Cambio Cosmic, Cambio Healthcare Systems, Linköping, Sweden) and the laboratory information system (ADBAKT, Autonik, Nyköping, Sweden). The data extraction was performed in four steps.

In the first step, all patients were identified who received a diagnosis code for pharyngotonsillitis (J02 or J03) from a PHCC or hospital clinic physician during the study period (Step 1, Fig. 2). Data regarding age, sex, RADTs, throat cultures, and antibiotic prescriptions from PHCCs and hospital clinics were then extracted and linked using the Swedish personal identification number and visit date. As the indication for antibiotic treatment could not be extracted from the EMR system, the following antibiotics relevant for treating pharyngotonsillitis in accordance with Swedish guidelines [1] were identified: phenoxymethylpenicillin (penicillin V), cefadroxil, and clindamycin. In addition, amoxicillin, erythromycin, and azithromycin were included as they are approved by the Swedish Medical Products Agency for treating pharyngotonsillitis. However, data were unavailable that would confirm whether patients collected their medication at a pharmacy or complied with prescribed treatment regime.

In the second step, patients who had at least one eligible visit to a PHCC with aetiological testing (see below) were selected (Step 2, Fig. 2). Five exclusion criteria for a visit were used: (1) visit date during the first 30 days of the study period; (2) a diagnosed pharyngotonsillitis or complication (defined as peritonsillitis, media otitis, sinusitis, lymphadenitis or sepsis, see Additional file 1: Table S1) the previous 30 days; (3) antibiotic prescription (as defined above) the previous 30 days; (4) a complication diagnosed on the same day as the visit; and (5) prescription of an antibiotic not indicated for a sore throat (Fig. 2). Aetiological testing was defined as an RADT for GAS performed on the same date as the visit or a throat culture performed within seven days. RADTs performed on the first day and cultures performed within a week were included because this routine mirrors clinical practice. Early descriptive analysis also revealed that most cultures were performed on the same day as the index visit, and an absolute majority within 7 days.

In the third step, a cohort (cohort 1) was formed with all patients from step 2 where an RADT had been performed (Step 3, Fig. 2). The first eligible visit for each patient was denoted as the index visit.

In the fourth step, using the same patients as in step 2, a new, explanatory cohort was created (cohort 2), with all patients who had been cultured (step 4, Fig. 2). As before, the first eligible visit with a culture was denoted as the index visit. As most patients had an RADT performed before they were cultured, many patients in cohort 2 were also in cohort 1, with common index visit dates.

In both cohorts, patients were grouped by antibiotic prescription on the day of their index visit, as early descriptive analysis revealed that most patients with a prescription were prescribed antibiotics during their index visit, whereas subsequent prescriptions were often made during a new visit, which we wanted to count as an outcome.

**Fig. 1:**
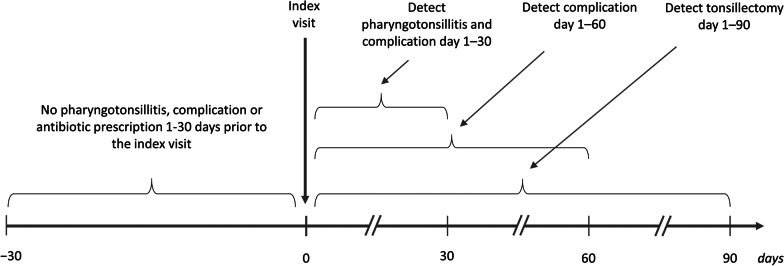
Observation time for patients with pharyngotonsillitis. In cohort 1 the index visit was defined as the first visit with a rapid antigen detection test (RADT) for group A streptococci (GAS) performed on the same day. In cohort 2, the index visit was defined as the first visit where a throat culture was performed within seven days. For a visit to be eligible there should neither be a visit for pharyngotonsillitis or a complication nor an antibiotic prescription during the last 30 days. In the follow-up, each patient was assessed for new visits for pharyngotonsillitis, a complication, and tonsillectomy up to 90 days from the index visit

Although the main criteria for inclusion in this study was a visit to a PHCC, the outcomes were defined as a visit to either a PHCC or a hospital clinic (Fig. 1). This was especially important for tonsillectomy, as it is never coded for in PHC, as well as for peritonsillitis, as these patients sometimes visit an emergency department at a hospital without first visiting a PHCC. If a patient had separate index visit dates for the two cohorts, the exclusion criteria made sure that no index visit would be registered as an outcome in the other cohort.

### Microbiological procedures

The RADT kit for GAS used in Kronoberg County during the study period was QuickVue Dipstick Strep A (Quidel Corporation, San Diego, CA, USA), a lateral-flow immunoassay using antibody-labelled particles [21]. The test detects viable and nonviable organisms directly from throat swabs.

Routine throat cultures for the recovery of large colony β-haemolytic streptococci used standard procedures, as previously described [9]. Starting in 2013, the laboratory also offered an extended throat culture that added an anaerobic plate for the recovery of *F.* *necrophorum* [9]. In late 2013, with the introduction of matrix-assisted laser desorption/ionization with time-of-flight mass spectrometer (MALDI-TOF), the reporting of streptococci transitioned from Lancefield classification to species identification. As a result, GAS was reported as *S.* *pyogenes* and most group C and G streptococci were reported as SDSE. In this study, group C or G streptococci were reported as SDSE. During the study period, before the transition, group C and G streptococci constituted 40% of all β-haemolytic streptococci in throat cultures; after the transition, the corresponding proportion for SDSE was 42% (data not shown).

### Statistical methods

Data were cleaned and analysed using Excel 2019 (Microsoft, Redmond, WA, USA) and SPSS 25.0 software (IBM, Armonk, NY, USA). Continuous variables with non-normal distribution or small sample sizes were reported as median (interquartile range, IQR). Categorical data were compared with two-sided Pearson χ^2^-test or Fisher’s exact test for independent groups, and McNemar test or Cochran’s Q test for dependent groups. A *p*-value < 0.05 was considered significant.

## Results

* For patients with multiple eligible visits, the first visit was denoted index visit. Due to double aetiological testing or multiple eligible visits, 1 127 patients were included in both cohorts.

### Study population

**Fig. 2:**
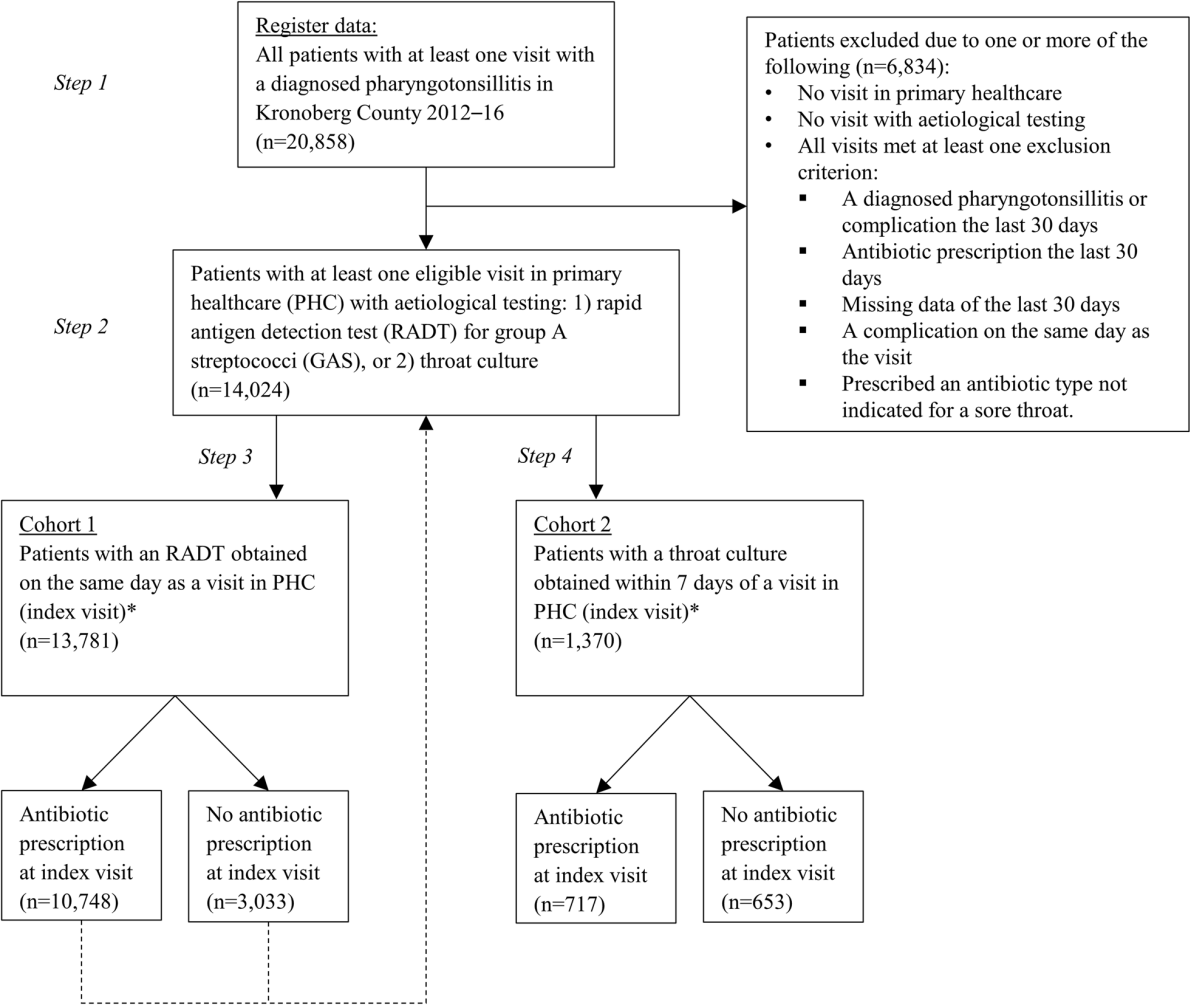
Flow chart of inclusion. All patients diagnosed with pharyngotonsillitis during a primary healthcare centre visit or hospital clinic visit in Kronoberg County during 2012–16 were selected from registry data (Step 1). From the group of patients with at least one eligible visit to a PHCC with aetiological testing (Step 2), two cohorts were created in turns: one based on rapid antigen detection testing (RADT) for group A streptococci (GAS) (Step 3), and one based on throat cultures (Step 4). As both cohorts were created from the same population, a patient could be included in both cohorts

Between 2012 and 2016, 20,858 patients were diagnosed with pharyngotonsillitis during at least one PHCC or hospital clinic visit. Of these, 14,024 had at least one eligible visit to a PHCC with aetiological testing, and from these patients two cohorts were formed (Fig. 2). Most index visits in cohort 1 and 2 took place during office hours (84% and 88%, respectively).

### Aetiology

In cohort 1, the RADT was positive for GAS in 9 170 patients (67%). In cohort 2, a regular culture was performed in 745 (54%) patients and an extended culture was performed in 625 (46%) patients. Of the 1 370 cultures registered within seven days of the index visit, 1 128 (82%) were performed on the same day as the index visit (Additional file 1: Table S2). Bacterial growth was found in 231 (31%) of the regular cultures and 234 (37%) of the extended cultures. Overall, GAS was detected in 201 (15%) patients and SDSE in 190 (14%) patients. *F.* *necrophorum* was detected in 95 (15%) patients who had extended cultures.

### Characteristics in relation to aetiology

GAS was most prevalent in children aged 0–14, with 80% of RADTs positive, whereas SDSE and *F.* *necrophorum* were most prevalent in patients aged 15–29. Table 1 lists the background characteristics of the patients in relation to aetiology.

**Table 1:** Characteristics of patients who performed a RADT for GAS or a throat culture

	RADT for GAS (cohort 1)			Throat culture (cohort 2)				
	Positive n = 9170	Negative n = 4611	All n = 13,781	*S. pyogenes*^1^ n = 201	*S. dysgalactiae* ssp. *equisimilis*^1^ n = 190	*F. necrophorum*^1,2^ n = 95	Negative n = 905	All^2^ n = 1 370
Female, n (%)	5 068 (55)	2 585 (56)	7 653 (56)	111 (55)	119 (63)	57 (60)	509 (56)	785 (57)
Age, years, median (IQR)	19 (7–36)	23 (16–38)	21 (9–37)	27 (12–38)	20 (17–29)	21 (17–26)	23 (17–38)	23 (17–36)
Age 0–14, n (%)	4 056 (44)	1 024 (22)	5 080 (37)	64 (32)	24 (13)	2 (2.1)	146 (16)	236 (17)
Age 15–29, n (%)	1 882 (21)	1 928 (42)	3 810 (28)	45 (22)	119 (63)	75 (79)	422 (47)	646 (47)
Age 30 + , n (%)	3 232 (35)	1 659 (36)	4 891 (35)	92 (46)	47 (25)	18 (19)	337 (37)	488 (36)
RADT performed (cohort 2), n (%)				139 (69)	154 (81)	73 (77)	660 (73)	1 011 (74)
RADT positive/all RADT, n (%)				104 (75)	6 (3.9)	6 (8.2)	83 (9.2)	196 (19)
Antibiotic treatment^3^, n (%)	8 751 (95)	1 997 (43)	10 748 (78)	151 (75)	97 (51)	52 (55)	429 (47)	717 (52)
Penicillin V, n (% of treated)	7 894 (90)	1 630 (82)	9524 (89)	106 (70)	73 (75)	34 (65)	324 (76)	527 (74)
Clindamycin, n (% of treated)	339 (3.9)	181 (9.1)	520 (4.8)	21 (14)	14 (14)	17 (33)	73 (17)	123 (17)
Cefadroxil, n (% of treated)	345 (3.9)	116 (5.8)	461 (4.3)	20 (13)	8 (8.2)	0	24 (5.6)	52 (7.3)
Other, n (% of treated)	173 (2.0)	70 (3.5)	243 (2.3)	4 (2.6)	2 (2.1)	1 (1.9)	8 (1.9)	15 (2.1)

Characteristics of patients who had a RADT for GAS performed on the same day as a visit to a primary healthcare centre or a culture performed to determine aetiology within seven days of a visit to a primary healthcare centre, in relation to aetiology

*RADT* Rapid Antigen Detection Test; *GAS* Group A streptococci (*S. pyogenes*)

^1^Refers to any finding (21 patients had a concomitant finding of two bacteria)

^2^To detect *F. necrophorum*, an extended culture was needed (see Methods section). In total, 625/1 370 (46%) of the patients had an extended culture

^3^Refers to antibiotics approved by the Swedish Medical Products Agency for treating pharyngotonsillitis (see Methods section) prescribed on the same day as the index visit. Antibiotic types are expressed as percentages of treated patients

### Frequency of outcomes

In the RADT cohort, 8.6% of the patients made a new visit for pharyngotonsillitis within 30 days (median = 12 days, IQR 4–16) and 1.6% made a new visit for a complication within 30 days (median = 12 days, IQR 3–21). Peritonsillitis accounted for 29% of these complications (median = 3 days, IQR 2–14) (Additional file 1: Table S3).

In the culture cohort, 20% of the patients made a new visit for pharyngotonsillitis within 30 days (median = 3 days, IQR 2–6) and 3.8% made a new visit for a complication within 30 days (median = 3 days, IQR 2–5). Peritonsillitis accounted for 78% of these complications (median = 2 days, IQR 2–5). Of the 51 patients with a complication, 61% were cultured on the same day as the complication, 35% were cultured at least one day before the complication, and two were cultured later.

### Antibiotics and outcomes

In the RADT cohort, in patients with a positive RADT pharyngotonsillitis within 30 days was less common in those who were prescribed antibiotics (8.7%; 95% CI 8.1–9.3%) than in those who were not prescribed antibiotics (12%; 95% CI 9.2–16%) (Table 2). In contrast, antibiotic prescription for patients with a negative RADT was associated with a higher proportion of pharyngotonsillitis within 30 days (9.7%; 95% CI 8.4–11%) compared to patients with no prescription (7.0%; 95% CI 6.1–8.0%). Antibiotic prescription was not associated with complication rates or tonsillectomy rates regardless of RADT result.

**Table 2:** Rapid Antigen Detection Test (RADT) for GAS result and antibiotic prescription in relation to outcomes

RADT for GAS^1^	Antibiotics^2^	Pharyngotonsillitis	Complication		Peritonsillitis^3^		Tonsillectomy
		30 d	30 d	60 d	30 d	60 d	90 d
Positive	All	791/8928 (8.9%)	136/8928 (1.5%)	211/8728 (2.4%)	33/8928 (0.37%)	37/8728 (0.42%)	23/8561 (0.27%)
	Antibiotics +	743/8528 (8.7%)	129/8528 (1.5%)	199/8338 (2.4%)	30/8528 (0.35%)	34/8338 (0.41%)	22/8182 (0.27%)
	Antibiotics −	48/400 (12%)	7/400 (1.8%)	12/390 (3.1%)	3/400 (0.75%)	3/390 (0.77%)	1/379 (0.26%)
	*p*	0.02	0.7	0.4	0.2†	0.2†	1†
Negative	All	369/4532 (8.1%)	78/4532 (1.7%)	104/4479 (2.3%)	30/4532 (0.66%)	34/4479 (0.76%)	13/4426 (0.29%)
	Antibiotics +	190/1965 (9.7%)	32/1965 (1.6%)	43/1939 (2.2%)	16/1965 (0.81%)	19/1939 (0.98%)	6/1918 (0.31%)
	Antibiotics −	179/2567 (7.0%)	46/2567 (1.8%)	61/2540 (2.4%)	14/2567 (0.55%)	15/2540 (0.59%)	7/2508 (0.28%)
	*p*	0.01	0.7	0.7	0.3	0.1	0.8

Rapid antigen detection test (RADT) for group A streptococci (GAS) and antibiotic prescription in relation to outcomes in patients where an RADT was performed on the same day as a visit to primary healthcare (n = 13 781)

^†^Fisher’s exact test

^1^Refers to RADTs performed on the same day as the index visit

^2^Refers to antibiotics approved by the Swedish Medical Products Agency for treating pharyngotonsillitis (see Methods section) prescribed on the same day as the index visit

^3^Patients with peritonsillitis are also included in “Complication”

In the culture cohort, antibiotics were prescribed to 717 (52%) patients on the same day as the index visit and to another 159 (12%) patients during the following seven days (Additional file 1: Table S4). Only 106 (7.7%) patients were prescribed an antibiotic before a sample for culture was obtained. In patients with SDSE antibiotic prescription was associated with a lower proportion of pharyngotonsillitis within 30 days compared with no prescription (Table 3). In contrast, in patients with a negative culture antibiotic prescription was associated with a larger proportion of pharyngotonsillitis and peritonsillitis within 30 days, compared with no prescription.

**Table 3:** Throat culture result and antibiotic prescription in relation to outcomes

Throat culture result^1^	Antibiotics^2^	Pharyngotonsillitis	Complication		Peritonsillitis^3^		Tonsillectomy
		30 d	30 d	60 d	30 d	60 d	90 d
*S. pyogenes*	All	30/190 (16%)	1/190 (0.53%)	3/188 (1.6%)	1/190 (0.53%)	1/188 (0.53%)	0/185
	Antibiotics +	21/143 (15%)	1/143 (0.70%)	2/142 (1.4%)	1/143 (0.70%)	1/142 (0.7%)	0/140
	Antibiotics −	9/47 (19%)	0/47	1/46 (2.2%)	0/47	0/46 (0%)	0/45
	*p*	0.5	1†	0.6†	1†	1†	-
*S. dysgalactiae* ssp. *equisimilis*^4^	All	37/171 (22%)	2/171 (1.2%)	2/170 (1.2%)	2/171 (1.2%)	2/170 (1.2%)	0/168
	Antibiotics +	13/87 (15%)	2/87 (2.3%)	2/86 (2.3%)	2/87 (2.3%)	2/86 (2.3%)	0/85
	Antibiotics −	24/84 (29%)	0/84 (0%)	0/84 (0%)	0/84 (0%)	0/84 (0%)	0/83
	*p*	0.03	0.5†	0.5†	0.5†	0.5†	-
*F. necrophorum*^*5*^	All	16/75 (21%)	9/75 (12%)	10/75 (13%)	8/75 (11%)	9/75 (12%)	4/72 (5.6%)
	Antibiotics +	11/41 (27%)	3/41 (7.3%)	4/41 (9.8%)	2/41 (4.9%)	3/41 (7.3%)	1/38 (2.6%)
	Antibiotics −	5/34 (15%)	6/34 (18%)	6/34 (18%)	6/34 (18%)	6/34 (18%)	3/34 (8.8%)
	*p*	0.2	0.3†	0.5†	0.1†	0.3†	0.3†
Negative (extended cultures only)	All	89/381 (23%)	22/381 (5.8%)	23/370 (6.2%)	19/381 (5.0%)	19/370 (5.1%)	9/363 (2.5%)
	Antibiotics +	56/194 (29%)	16/194 (8.2%)	17/188 (9.0%)	15/194 (7.7%)	15/188 (8.0%)	4/184 (2.2%)
	Antibiotics −	33/187 (18%)	6/187 (3.2%)	6/182 (3.3%)	4/187 (2.1%)	4/182 (2.2%)	5/179 (2.8%)
	*p*	0.01	0.04	0.02	0.01	< 0.001	0.8

Antibiotic prescription and results from throat cultures performed within seven days from the index visit for pharyngotonsillitis in relation to outcomes (n=1 370)

^†^Fisher’s exact test

^1^Refers to findings of single pathogens within seven days of the index visit

^2^Refers to antibiotics approved by the Swedish Medical Products Agency for treating pharyngotonsillitis (see Methods section) prescribed on the same day as the index visit

^3^All cases with peritonsillitis are also included in “Complication”

^4^Before 2013, *S. dysgalactiae* ssp.* equisimilis* was reported as either group C or G streptococci, which is detailed in the Methods section

^5^To detect* F. necrophorum*, an extended culture was needed (see Methods section). In total, 625/1 370 (46%) of the patients had an extended culture

In the RADT cohort, the proportion of peritonsillitis within 30 days differed with antibiotic chosen both in patients with a positive RADT and a negative RADT, with the lowest proportions among patients who were prescribed penicillin V (Table 4). In the culture cohort, antibiotic type was not associated with the outcomes.

**Table 4:** Aetiological test results and antibiotic choice in relation to outcomes

Aetiological test	Antibiotics^1^	Pharyngotonsillitis	Complication		Peritonsillitis^2^		Tonsillectomy
		30 d	30 d	60 d	30 d	60 d	90 d
RADT for GAS							
Positive	PcV	652/7685 (8.5%)	108/7685 (1.4%)	169/7504 (2.3%)	23/7685 (0.30%)	27/7504 (0.36%)	16/7358 (0.22%)
	Clindamycin	37/333 (11%)	10/333 (3.0%)	14/331 (4.2%)	4/333 (1.2%)	4/331 (1.2%)	3/328 (0.91%)
	Cefadroxil	31/340 (9.1%)	5/340 (1.5%)	7/335 (2.1%)	2/340 (0.59%)	2/335 (0.60%)	1/331 (0.30%)
	*p*	0.2	0.07†	0.06	0.02†	0.04†	0.04†
Negative	PcV	145/1604 (9.0%)	25/1604 (1.6%)	33/1582 (2.1%)	10/1604 (0.62%)	13/1582 (0.82%)	3/1564 (0.19%)
	Clindamycin	25/178 (14%)	5/178 (2.8%)	6/176 (3.4%)	5/178 (2.8%)	5/176 (2.8%)	2/174 (1.1%)
	Cefadroxil	13/115 (11%)	1/115 (0.87%)	2/115 (1.7%)	0/115	0/115	1/115 (0.87%)
	*p*	0.08	0.4†	0.4†	0.02†	0.046†	0.052†
Throat culture^3^							
*S. pyogenes*	PcV	15/101 (15%)	1/101 (0.99%)	1/100 (1.0%)	1/101 (0.99%)	1/100 (1.0%)	0/98
	Clindamycin	3/19 (16%)	0/19	0/19	0/19	0/19	0/19
	Cefadroxil	3/19 (16%)	0/19	1/19 (5.3%)	0/19	0/19	0/19
	*p*	1†	1†	0.5†	1†	1†	-
*S. dysgalactiae* ssp. *equisimilis*^4^	PcV	10/64 (16%)	2/64 (3.1%)	2/63 (3.2%)	2/64 (3.1%)	2/63 (3.2%)	0/62
	Clindamycin	3/13 (23%)	0/13	0/13	0/13	0/13	0/13
	Cefadroxil	0/8	0/8	0/8	0/8	0/8	0/8
	*p*	0.5†	1†	1†	1†	1†	-
*F. necrophorum*^*5*^	PcV	7/25 (28%)	1/25 (4.0%)	2/25 (8.0%)	0/25	1/25 (4.0%)	1/22 (4.5%)
	Clindamycin	4/15 (27%)	2/15 (13%)	2/15 (13%)	2/15 (13%)	2/15 (13%)	0/15
	Cefadroxil	0/0	0/0	0/0	0/0	0/0	0/0
	*p*	1†	0.6†	0.6†	0.1†	0.6†	1†
Negative	PcV	83/318 (26%)	20/318 (6.3%)	23/311 (7.4%)	17/318 (5.3%)	18/311 (5.8%)	4/307 (1.3%)
	Clindamycin	17/70 (24%)	6/70 (8.6%)	7/70 (10%)	6/70 (8.6%)	6/70 (8.6%)	2/69 (2.9%)
	Cefadroxil	9/24 (38%)	0/24	0/24	0/24	0/24	0/23
	*p*	0.4	0.4†	0.3†	0.3†	0.4†	0.5†

^†^Fisher’s exact test;* RADT* Rapid Antigen Detection Test;* GAS* Group A Streptococci

^1^Antibiotics prescribed on the same day as the index visit. In Sweden, PcV is the recommended antibiotic for pharyngotonsillitis and Clindamycin and Cefadroxil are alternatives. All antibiotics are recommended for ten days of treatment

^2^All cases with peritonsillitis are also included in “Complication”

^3^Refers to findings of single pathogens within seven days of the index visit

^4^Before 2013, *S. dysgalactiae* ssp.* equisimilis* was reported as either group C or G streptococci, which is detailed in the Methods section

^5^To detect* F. necrophorum*, an extended culture was needed (see Methods section). In total, 625/1 370 (46%) of the patients had an extended culture

## Discussion

In this registry-based study of patients diagnosed with pharyngotonsillitis at a visit in primary healthcare, antibiotic prescription was associated with a lower proportion of return visits for pharyngotonsillitis in patients with a positive RADT for GAS but with a higher proportion of return visits in patients with a negative RADT for GAS. Regardless of test result, antibiotic prescription was not associated with a reduced incidence of purulent complications.

### Meaning of the study

With RADTs being positive in 67% of tested patients (i.e. 47% of the whole population studied), GAS was the most common aetiology in our material. This proportion is much higher than expected from prevalence studies [3, 9, 22] and probably points to a classification bias, where the choice of diagnosis codes might have been affected by the test result. In throat cultures, all detected bacteria were equally common, but this finding is hard to interpret as the reason for obtaining a sample for culture (e.g. a more severe clinical presentation) is unknown. Moreover, a positive RADT should reduce the diagnostic necessity of a culture, so the true prevalence of GAS could be underestimated. The prevalence (15%) of *F.* *necrophorum* in prolonged anaerobic culture suggests that a similar proportion of routine cultures for streptococci might also harbour *F.* *necrophorum*. Certainly, the clinical presentation could have led to a selection bias of extended cultures; however, recent meta-analyses have reported a 18–19% prevalence of *F.* *necrophorum* in patients with a sore throat diagnosed in PHC [e, 12]. In our study, *F.* *necrophorum* and SDSE were most prevalent among patients aged 15–29. This finding is in line with previous studies: a low prevalence of *F.* *necrophorum* and SDSE in children and the highest prevalence in adolescents and young adults [8–11, 23]. Conversely, GAS was most prevalent in children, which probably reflects a large proportion of carriage in this age group [22, 24].

There was an association between antibiotic prescription and fewer return visits for pharyngotonsillitis in patients with a positive RADT, suggesting a protective role for antibiotics. This finding contrasts with previous findings of increased re-attendance in patients prescribed an immediate antibiotic due to changed expectations and behaviour (i.e., “medicalisation”) [25–27]. On the other hand, antibiotics were associated with a higher rate of return visits for pharyngotonsillitis in patients with a negative RADT, which may suggest that the treatment was not effective for this group or that there was a medicalising effect.

Although the culture cohort only constituted a small proportion of all patients, there was an association between antibiotic prescription and fewer return visits for pharyngotonsillitis in patients with SDSE, suggesting a protective role of antibiotics in a subset of the patients with a negative RADT. Surprisingly, patients with negative cultures and antibiotic prescription had a higher incidence of all outcomes measured regardless of the antibiotic used for treatment. Our first thought was that most of these patients had initiated antibiotic treatment before being cultured, but this was only the case in 10% of the patients. Other explanations might be medicalisation, ineffective antibiotics, and confounding by indication (i.e., patients with more severe illness are more likely to receive antibiotics) [28].

Antibiotic prescription was not associated with fewer complications in any cohort. However, complications, especially peritonsillitis, are rare outcomes, and since 95% of the patients with a positive RADT were prescribed antibiotics, the comparison group was rather small. The actual numbers did point to a protective role for antibiotics for complications in patients with a positive RADT (Table 2), but there might have been too few cases to detect a significant difference. The small numbers were also evident in the culture cohort as almost none of the comparisons, no matter how large the difference, were statistically significant. The complication rate in this study was similar to a previous registry study in PHC [29] but lower than the average in randomised controlled trials [5]. Previous studies on the protective role of antibiotics are somewhat conflicting, with the limited trial evidence suggesting a lowered relative risk (RR = 0.10, 95% CI = 0.01–0.79, in studies conducted after the 1950s) [5], but large recent observational studies suggest either no protective role [17, 29] or a very small absolute risk reduction with a huge Number needed to treat (NNT) [30, 31].

Most patients who developed peritonsillitis were diagnosed within a few days after inclusion, with a median of three days in the RADT cohort and two days in the culture cohort. In the long-term follow-up, almost no new cases emerged between 30 and 60 days. These findings are consistent with previous reports of a very fast onset of peritonsillitis [17, 29, 32, 33], suggesting that some of the cases of peritonsillitis might already have been imminent or misdiagnosed as pharyngotonsillitis at inclusion.

Most treated patients received penicillin V, but the overall picture was that the antibiotic chosen was unrelated to the outcomes, a finding in line with a previous meta-analysis [34]. The exception was peritonsillitis and tonsillectomy in the RADT cohort, where penicillin V was associated with fewer cases both in patients with a positive RADT and in patients with a negative RADT. In Sweden, patients with recurring pharyngotonsillitis are generally required to have tried three types of antibiotics before being eligible for tonsillectomy; therefore, clindamycin and cefadroxil, which are second-choice antibiotics, might be associated with complications and tonsillectomy more than penicillin V.

### Strengths and weaknesses of the study

To our knowledge, this is the largest registry-based study investigating pharyngotonsillitis in PHC, with almost complete data on all recorded diagnoses of pharyngotonsillitis, complications and tonsillectomies for five years, from PHC (office hours and out-of-hours) and hospital clinics. In addition, this is the first study to present data on patients who had *F.* *necrophorum* detected in routine cultures. Unlike case–control studies and case reports, this study followed a cohort of patients with pharyngotonsillitis prospectively to estimate the incidence of outcomes. As no randomised controlled trial has been sufficiently sized to study the effect of antibiotics on non-group A streptococci and *F.* *necrophorum* in patients with pharyngotonsillitis, this study offers valuable observational data.

However, a registry study comes with inherent weaknesses. For example, we did not know the clinical circumstances of the patients (e.g. severity and duration of symptoms, patients’ expectations, and physicians’ intentions with tests and antibiotic prescription), differential diagnostic reasoning, and inter-rater reliability in terms of diagnostic skills and coding. Therefore, all results are based on the factual codes, test results, and prescriptions registered in the EMR system, a circumstance that calls for a cautious interpretation of the results. On the other hand, this study is based on a large quantity of real-life clinical data from PHC, mirroring both the disease panorama and the behaviour of physicians, nurses, and patients rather than on experimental trial data on a small, selected, and closely monitored population.

The definition of pharyngotonsillitis was confined to the applicable codes in ICD-10 (J02.x and J03.x) although we know from clinical experience and previous studies [35] that sore throats are sometimes coded as “upper respiratory infection” or “viral infection”, especially if the patient has compelling viral symptoms. We made this choice because sore throat as a symptom does not lend itself to registry-based studies, and other codes encompass too many conditions to be useful. Narrowing in on ICD codes for pharyngotonsillitis, however, might have selected a population with a higher likelihood to benefit from antibiotics.

Excluding patients with a diagnosed complication on the same day might have underestimated the complication rate of certain bacteria. However, the primary aim was not to establish a link between aetiology and complications but to follow patients with a pharyngotonsillitis in PHC and study the effect of antibiotic prescription on different outcomes. Another study, focusing on complications, especially peritonsillitis, is nonetheless fully possible with this database and already in the planning.

### Unanswered questions and future research

To better appreciate the effect of antibiotic treatment on resolution of symptoms, relapses, and complications in patients with non-group A streptococcal bacterial aetiology, a sufficiently sized randomised controlled trial is warranted. As regular penicillin V was found to be non-inferior to clindamycin and cefadroxil in this study, it might then be an interesting candidate to investigate further. The prevalence of throat cultures was low in our material, and any subsequent registry study on this topic will need to consider this in sizing calculations.

## Conclusions

Antibiotic prescription was associated with a lower proportion of return visits for pharyngotonsillitis in patients with a positive RADT for GAS but with a higher proportion of return visits in patients with a negative RADT. Antibiotic prescription was not associated with a reduced incidence of purulent complications regardless of test result. Routine throat cultures were sparse in our setting (in line with national guidelines) and too few to draw any strong conclusions about the possible divergent outcomes in patients positive for SDSE and/or *F.* *necrophorum*.

## Supplementary Information

**Additional file 1.** Additional tables.

## Data Availability

The datasets used and/or analysed during the current study are available from the corresponding author on reasonable request.

## Abbreviations

GAS: Group A streptococci
EMR: Electronic medical record
IQR: Interquartile range
PHC: Primary healthcare
PHCC: Primary healthcare centres
RADT: Rapid antigen detection test
SDSE: *Streptococcus dysgalactiae* Subspecies *equisimilis*
