# Supplementary material for: Association between bacterial finding, antibiotic treatment and clinical course in patients with pharyngotonsillitis: a registry-based study in primary healthcare in Sweden
Source: BMC Infect Dis. 2021 Aug 9;21:779. doi: 10.1186/s12879-021-06511-y (PMC8351112; doi:10.1186/s12879-021-06511-y)
Supplement: Supplementary file 1 — Additional file 1. Additional tables. [file 12879_2021_6511_MOESM1_ESM.docx]

# Supplementary material

**Supplementary Table 1. List of ICD-codes in data retrieval**

| **Title of data** | | | |
| --- | --- | --- | --- |
| Codes selected for data retrieval in the register-based study of pharyngotonsillitis in Kronoberg County 2012-16. | | | |
|  |  |  |  |
| **Description of data** | | | |
| The column “Diagnosis code” shows ICD-10 codes. | | | |
| The column “Description” is the standard English description for clarity (not used in Sweden). | | | |
| The column “Diagnosis code, primary care” shows the standard Swedish primary care codes according to a simplified version of ICD-10 (KSH-97). | | | |
| The column “Description, primary care” is the standard English description for clarity (not used in Sweden). | | | |
| The heading "Intervention code" refers to the national Classification of Health Interventions, or "KVÅ" ("Klassifikation av vårdåtgärder"). | | | |
|  |  |  |  |
| **Diagnosis code** | **Description** | **Diagnosis code, primary healthcare** | **Description, primary healthcare** |
| J02- | Acute pharyngitis | J02- | Acute pharyngitis |
| J020 | Streptococcal pharyngitis | J02- | Acute pharyngitis |
| J028 | Acute pharyngitis due to other specified organisms | J02- | Acute pharyngitis |
| J029 | Acute pharyngitis, unspecified | J02- | Acute pharyngitis |
| J03- | Acute tonsillitis | J03- | Acute tonsillitis |
| J030 | Streptococcal tonsillitis | J03- | Acute tonsillitis |
| J038 | Acute tonsillitis due to other specified organisms | J03- | Acute tonsillitis |
| J039 | Acute tonsillitis, unspecified | J03- | Acute tonsillitis |
| J36- | Peritonsillar abscess | J36- | Peritonsillar abscess |
| J369 | Peritonsillar abscess | J36- | Peritonsillar abscess |
|  |  |  |  |
| J01- | Acute sinusitis | J01- | Acute sinusitis |
| J010 | Acute maxillary sinusitis | J01- | Acute sinusitis |
| J011 | Acute frontal sinusitis | J01- | Acute sinusitis |
| J012 | Acute ethmoidal sinusitis | J01- | Acute sinusitis |
| J014 | Acute pansinusitis | J01- | Acute sinusitis |
| J018 | Other acute sinusitis | J01- | Acute sinusitis |
| J019 | Acute sinusitis, unspecified | J01- | Acute sinusitis |
|  |  |  |  |
| H660 | Acute suppurative otitis media | H660 | Acute suppurative otitis media |
| H664 | Suppurative otitis media, unspecified | H669P | Otitis media, unspecified |
| H669 | Otitis media, unspecified | H669P | Otitis media, unspecified |
| H669P | Otitis media, unspecified | H669P | Otitis media, unspecified |
|  |  |  |  |
| H70- | Mastoiditis and related conditions | H70- | Mastoiditis |
| H700 | Acute mastoiditis | H70- | Mastoiditis |
| H701 | Chronic mastoiditis | H70- | Mastoiditis |
| H709 | Mastoiditis, unspecified | H70- | Mastoiditis |
| H750 | Mastoiditis in infectious and parasitic diseases classified elsewhere | H939P | Other disorders of ear |
|  |  |  |  |
| L04- | Acute lymphadenitis | L04- | Acute lymphadenitis |
| L040 | Acute lymphadenitis of face, head and neck | L04- | Acute lymphadenitis |
| L048 | Acute lymphadenitis of other sites | L04- | Acute lymphadenitis |
| L049 | Acute lymphadenitis, unspecified | L04- | Acute lymphadenitis |
|  |  |  |  |
| A41-P | Other sepsis | A41-P | Septicaemia |
| A419 | Sepsis, unspecified | A41-P | Septicaemia |
| A418 | Other specified sepsis | A41-P | Septicaemia |
| A400 | Sepsis due to streptococcus, group A | A41-P | Septicaemia |
| A414 | Sepsis due to streptococcus, group A | A41-P | Septicaemia |
| A408 | Other streptococcal sepsis | A41-P | Septicaemia |
| A409 | Streptococcal sepsis, unspecified | A41-P | Septicaemia |
|  |  |  |  |
| M726 | Necrotizing fasciitis | M799P | Other soft tissue disorders |
|  |  |  |  |
| **Intervention code** |  |  |  |
| EMB10 | Tonsillectomy |  |  |

**Supplementary Table 2. Number of days from index visit to obtained samples for throat culture**

|  | Regular cultures | |  | Extended cultures | |  | All cultures | |
| --- | --- | --- | --- | --- | --- | --- | --- | --- |
| Days from index visit to culture | n (%) | Cumulative percent |  | n (%) | Cumulative percent |  | n (%) | Cumulative percent |
| 0 | 640 (86) | 85.9 |  | 488 (78) | 78.1 |  | 1 128 (82) | 82.3 |
| 1 | 33 (4.4) | 90.3 |  | 38 (6.1) | 84.2 |  | 71 (5.2) | 87.5 |
| 2 | 25 (3.4) | 93.7 |  | 26 (4.2) | 88.3 |  | 51 (3.7) | 91.2 |
| 3 | 15 (2.0) | 95.7 |  | 21 (3.4) | 91.7 |  | 36 (2.6) | 93.9 |
| 4 | 9 (1.2) | 96.9 |  | 18 (2.9) | 94.6 |  | 27 (2.0) | 95.8 |
| 5 | 8 (1.1) | 98 |  | 10 (1.6) | 96.2 |  | 18 (1.3) | 97.2 |
| 6 | 5 (0.67) | 98.7 |  | 14 (2.2) | 98.4 |  | 19 (1.4) | 98.5 |
| 7 | 10 (1.3) | 100 |  | 10 (1.6) | 100 |  | 20 (1.5) | 100 |
| Total | 745 (100) |  |  | 625 (100) |  |  | 1 370 (100) |  |

**Supplementary Table 3. Frequency of complications within 30 days after a pharyngotonsillitis in primary healthcare**

|  | RADT for GAS (cohort 1) | |  | Throat culture (cohort 2) | |
| --- | --- | --- | --- | --- | --- |
|  | n=13 781 | |  | n=1 370 | |
| Complication | n (%) | % of all complications |  | n (%) | % of all complications |
| Peritonsillitis | 63 (0.47) | 29 |  | 40 (2.9) | 78 |
| Media otitis | 100 (0.74) | 47 |  | 3 (0.22) | 5.9 |
| Sinusitis | 44 (0.33) | 21 |  | 2 (0.15) | 3.9 |
| Lymphadenitis | 6 (0.045) | 2.8 |  | 6 (0.44) | 12 |
| Sepsis | 1 (0.0074) | 0.47 |  | - | - |
| Total | 214 (1.6) | 100 |  | 51 (3.7) | 100 |

**Supplementary Table 4. Type of throat culture and number of days from index visit to antibiotic prescription**

|  | Patients with a regular culture (n=745) | |  | Patients with an extended culture (n=625) | |  | Patients with any culture (n=1 370) | |
| --- | --- | --- | --- | --- | --- | --- | --- | --- |
| Days from index visit to prescription | n (%) | Cumulative percent |  | n (%) | Cumulative percent |  | n (%) | Cumulative percent |
| 0 | 372 (82) | 81.6 |  | 345 (82) | 82.1 |  | 717 (82) | 81.8 |
| 1 | 26 (5.7) | 87.3 |  | 25 (6.0) | 88.1 |  | 51 (5.8) | 87.7 |
| 2 | 32 (7.0) | 94.3 |  | 15 (3.6) | 91.7 |  | 47 (5.4) | 93 |
| 3 | 9 (2.0) | 96.3 |  | 11 (2.6) | 94.3 |  | 20 (2.3) | 95.3 |
| 4 | 7 (1.5) | 97.8 |  | 9 (2.1) | 96.4 |  | 16 (1.8) | 97.1 |
| 5 | 2 (0.4) | 98.2 |  | 9 (2.1) | 98.6 |  | 11 (1.3) | 98.4 |
| 6 | 3 (0.7) | 98.9 |  | 4 (1.0) | 99.5 |  | 7 (0.8) | 99.2 |
| 7 | 5 (1.1) | 100.0 |  | 2 (0.5) | 100 |  | 7 (0.8) | 100 |
| Total number of patients with a prescription within 7 days | 456 (100) |  |  | 420 (100) |  |  | 876 (100) |  |
